# Light-Driven Catalytic Activity of Green-Synthesized SnO_2_/WO_3–*x*_ Hetero-nanostructures

**DOI:** 10.1021/acsomega.3c02330

**Published:** 2023-05-24

**Authors:** Faroha Liaqat, Urwa tul Vosqa, Fatima Khan, Abdul Haleem, Mohammed Rafi Shaik, Mohammed Rafiq H. Siddiqui, Mujeeb Khan

**Affiliations:** † Department of Chemistry, Quaid-i-Azam University, 45320 Islamabad, Pakistan; ‡ CAS Key Laboratory of Soft Matter Chemistry, Department of Polymer Science and Engineering, University of Science and Technology of China, Hefei, Anhui 230026, China; § Department of Chemistry, College of Science, King Saud University, P.O. Box 2455, Riyadh 11451, Saudi Arabia; ∥ Department of Chemistry, University of Liverpool, Liverpool L69 7ZD, United Kingdom

## Abstract

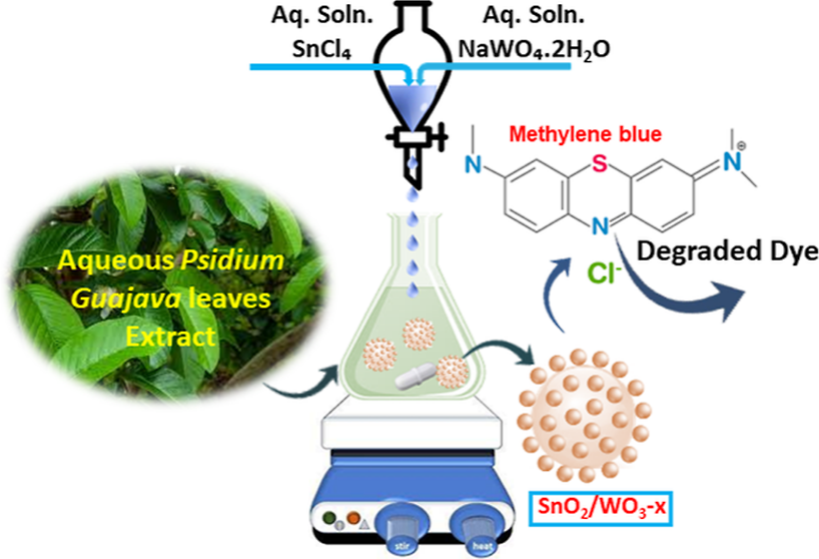

This work reports an environmentally friendly and economically feasible green synthesis of monometallic oxides (SnO_2_ and WO_3_) and their corresponding mixed metal oxide (SnO_2_/WO_3–*x*_) nanostructures from the aqueous *Psidium guajava* leaf extract for light-driven catalytic degradation of a major industrial contaminant, methylene blue (MB). *P. guajava* is a rich source of polyphenols that acts as a bio-reductant as well as a capping agent in the synthesis of nanostructures. The chemical composition and redox behavior of the green extract were investigated by liquid chromatography–mass spectrometry and cyclic voltammetry, respectively. Results acquired by X-ray diffraction and Fourier transform infrared spectroscopy confirm the successful formation of crystalline monometallic oxides (SnO_2_ and WO_3_) and bimetallic SnO_2_/WO_3–*x*_ hetero-nanostructures capped with polyphenols. The structural and morphological aspects of the synthesized nanostructures were analyzed by transmission electron microscopy and scanning electron microscopy coupled with energy-dispersive X-ray spectroscopy. Photocatalytic activity of the synthesized monometallic and hetero-nanostructures was investigated for the degradation of MB dye under UV light irradiation. Results indicate a higher photocatalytic degradation efficiency for mixed metal oxide nanostructures (93.5%) as compared to pristine monometallic oxides SnO_2_ (35.7%) and WO_3_ (74.5%). The hetero-metal oxide nanostructures prove to be better photocatalysts with reusability up to 3 cycles without any loss in degradation efficiency or stability. The enhanced photocatalytic efficiency is attributed to a synergistic effect in the hetero-nanostructures, efficient charge transportation, extended light absorption, and increased adsorption of dye due to the enlarged specific surface area.

## 1 Introduction

Major causes of water pollution in the developing world^1^ are industrial effluents such as dyes, use of excessive pesticides, fertilizers, and insecticides, and oil spills. Textile dyeing contributes 17–20% toward water pollution where a majority of dyes do not bind to the substrate (fibers) and are released into water untreated, leading to water pollution.^2,3^ Methylene blue (MB) dye is a major industrial effluent because of its excessive usage in textile and leather industries, toxicity, and water-soluble nature.^4−6^ Conventional methods to degrade the MB dye pose a challenge owing to its aromatic structure, hydrophilic nature, and high stability against light and temperature.^7,8^ Up to now, various strategies have been adopted to minimize the impact of hazardous dyes and organic compounds contributing toward water contamination, such as physical adsorption,^9^ ion exchange,^10^ coagulation,^11^ chlorination,^12^ and anaerobic and aerobic treatment methods.^13,14^ However, the high processing cost, non-eco-friendly approach, and sludge formation have been some drawbacks associated with these conventional methods.^15,16^ Advanced oxidation processes (AOPs) provide a viable alternative^17,18^ for the complete degradation of carcinogenic and bio-resistant pollutants through chemical oxidation processes involving highly reactive radical species (e.g., OH^•^ and O_2_^•^).^19,20^ Major advantages of AOPs are their environmental-friendly nature and almost no production of secondary toxic compounds as byproducts during oxidation, since the reactive radicals with a high oxidation potential can oxidize most of the organic compounds to CO_2_ and H_2_O.^21^ Photocatalysis offers an attractive prospect of using inexpensive photochemically stable photocatalysts (such as semiconductors) in sunlight or UV irradiation for effective degradation of dyes under ambient conditions. The key concerns in this regard are the (i) production of electrons (e^–^) and holes (h^+^) on irradiation, (ii) energy band gap of the photocatalyst,^22^ and (iii) effective separation of e^–^/h^+^ pairs.^23^

Homogeneous inorganic-based photocatalysts,^24,25^ though well-explored, suffer from the problem of metal-ion leaching, thus leading to secondary pollution. On the other hand, heterogeneous photocatalysts prove more advantageous due to their easy processability, mild degradation conditions, high efficiency, and versatile character, in addition to the ease of recycling.^26,27^ In this context, metal/semiconductor oxide nanocrystals prove to be efficient photocatalysts for the treatment of water contaminants due to their potent physiochemical properties, such as a high surface area, porosity, large number of dangling bonds, high carrier capacity, selectivity, long life span, high photosensitivity, and strong oxidizing activity.^28,29^ An effectual photocatalyst would have a band gap compatible for efficient utilization of solar energy with a lower charge recombination rate.^30^ Effective photocatalyst modification strategies have included doping,^31,32^ the use of a porous support with a large surface area,^33^ and sensitization of the semiconductors by dyes or quantum dots to obtain high photodegradation efficiency.^34^ Next-generation photoactive materials have been developed by investigation on heterostructures^35^ having a low band gap, an extended range of optical absorption spectrum, and greater separation of photoinduced (e^–^/h^+^) and enhanced photocatalytic activity due to the synergistic effect.^36,37^ In particular, bimetallic nanostructures having two distinct metals/metal oxides in one phase^38^ show enhanced properties compared to monometallic nanostructures due to their increased functionality.^39^ In addition, other properties such as stability, selectivity, catalytic activity,^40,41^ and tunability that are highly reliant on the size and shape of nanoparticles can also be improved by altering the composition of corresponding constituents in bimetallic nanostructures.^42^ Depending on the method of preparation and metal distribution, bimetallics are vaguely classified into alloys and core–shell structures,^43,44^ while in terms of atomic arrangement, these are classified into four categories^45−47^ of alloys, intermetallics, sub-clusters, and core–shell.

Most of the available photocatalysts have been developed by synthetic routes that require aggressive reducing agents, toxic solvents, and non-decomposable stabilizers and are expensive and demand harsh conditions.^38^ Moreover, the associated chemical reactions lead to secondary pollution due to the formation of perilous byproducts.^48^ Green synthesis provides an alternative route due to its eco-friendly nature, cost efficiency, mild conditions, and no specific requirement and perilous reducing or stabilizing agents.^49^ Bio-resources, such as micro-organisms, plants, biomass, amino acids, enzymes, and so forth, have been utilized in the synthesis of nanostructures,^50,51^ making this method eco-friendly, economical, and sustainable.^52^ In particular, plant extracts contain many versatile phytochemicals, such as carotenoids, quercetin, flavonoids, alkaloids, glycosides, polyphenolic acids, and quinones, and other bioactive compounds which are involved in the reduction, synthesis, as well as stabilization of nanostructures.^52^

**Figure 1:**
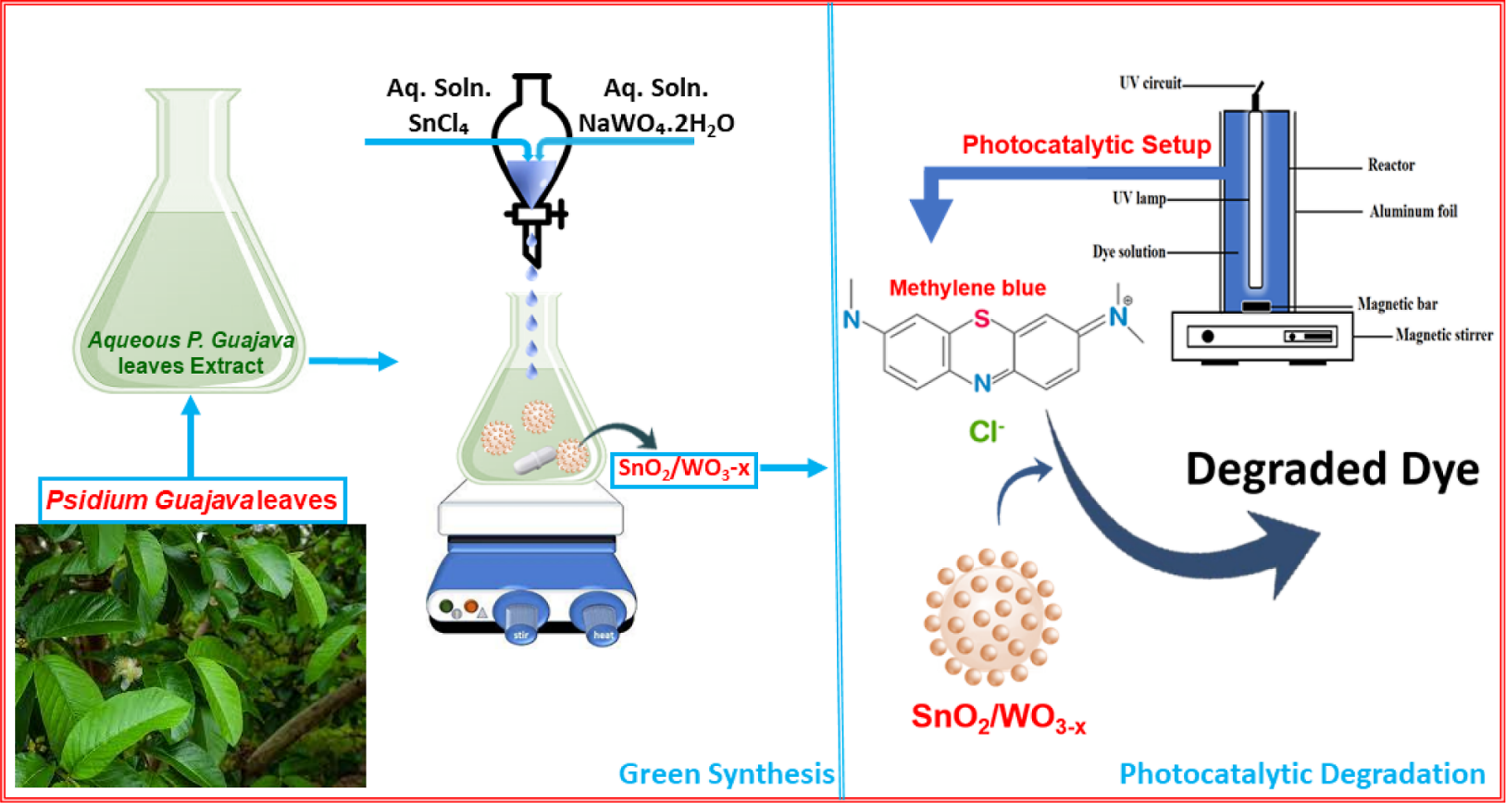
Schematic of the green route employed for the synthesis of the SnO_2_/WO_3–*x*_ hetero-nanostructure and its photocatalytic application.

This research work emphasizes an environmentally friendly way to synthesize efficient monometallic oxide nanocrystals of SnO_2_ and WO_3_ and their hetero-oxide counterparts (SnO_2_/WO_3–*x*_) as efficient photocatalysts using the *Psidium guajava* (guava plant) leaf extract for the first time for the degradation of MB dye. The choice of monometallic and bimetallic nanostructures has been carefully made keeping in mind the proven photocatalytic record of tungsten oxide, attributed to its tunable band gap and extensive absorption, chemical reactivity, and electrochromic properties.^53,54^ Tin oxide nanoparticles can also act as a suitable photocatalyst^55^ due to its n-type nature, transparent character in the visible spectrum, variable oxidation states and thermal stability, and fast electron transportation on light irradiation because of elevated electron mobility. In this work, we report a new eco-friendly green route for the synthesis of hetero-oxide SnO_2_/WO_3–*x*_ nanostructures using the leaf extract of *P. guajava* with the objective of obtaining efficient photocatalysts showing extended light absorption (Figure 1), enhanced charge separation, and improved photocatalytic performance in the degradation of MB dye, a common industrial pollutant, due to the synergistic effect observed in bimetallic nanostructures. Besides, the photocatalytic properties of nanostructured bimetallic oxides are compared with their monometallic metal oxide counterparts.

## 2 Results and Discussion

### 2.1 Role of Biomolecules in the Green Synthesis and Stabilization of Nanostructures: LC–MS and CV Analyses

The synthetic green route is designed to preserve the characteristic benefits of green chemistry, such as eco-friendliness, non-toxicity, and an easy one-pot synthesis. To this purpose, the reducing and stabilizing components of the *P. Guajava* leaf extract have been extracted in deionized (DI) water with high yields. During synthesis, the biomolecules act as the capping agent on the synthesized metal oxide nanostructures, as the nucleation process is followed by growth under controlled conditions.

The LC–MS analysis of the green extract was performed to identify the biomolecules, such as polyphenols, which can act as stabilizing agents in the synthesis of nanostructures.^56,57^ The recorded data were compared to the academic literature^58−60^ and the Global Natural Products Social Molecular Networking (GNPS) database in order to identify the phytochemicals present in the green extract, which are listed in Table 1.

**Table 1:** Major Biomolecules Identified by LC–MS Analysis of the *P. guajava* Leaf Extract with Retention Time and Name of the Compound

sr. no.	*m*/*z*	retention time (min)	identified compound
1.	380.0	3.15	tetrahydroxystilbene galloyl hexoside derivative
2.	423.0	3.60	guava-coumaric acid derivative
3.	499.3	4.48	HHDP glucose isomer
4.	535.0	4.85	guavinosideA (benzophenone glycoside)
5.	556.0	5.10	tetrahydroxystilbene galloyl hexoside
6.	622.5	5.81	guava-coumaric acid isomer
7.	643.7	6.10	quercetin galloylhexoside isomer derivative
8.	660.0	6.22	quercetin galloylhexoside isomer
9.	676.0	6.40	guavinB
10.	710.0	6.80	geranin isomer derivative
11.	935.7	9.25	casuarinin/casuarictin isomer (bis-HHDP galloyl glucose)
12.	953.0	9.42	geranin isomer

The mass chromatographs indicate the major entities present in the *P. guajava* extract in large amounts (Figure S1) that may prevent agglomeration of the synthesized nanostructures by acting as capping agents, thereby also acting as size-controllers. Table 1 shows the identified biomolecules with their retention times, and a careful observation points toward the presence of polyphenols and flavonoids. For example, the compound tetrahydroxystilbene galloyl hexoside (sr. 5) exhibits a molecular ion peak at *m*/*z* 556, while the fragment ion at *m*/*z* 380 (sr. 1) is attributed to tetrahydroxystilbene galloyl after the loss of a hexosyl residue. Similarly, the guava-coumaric acid isomer derivative (sr. 6, *m*/*z* 622.5) and its fragment ion (sr. 2, *m*/*z* 423) after the loss of a *p*-coumaroyl or feruloyl residues can clearly be identified. Additionally, there is evidence of the presence of glycoside (sr. 4, *m*/*z* 535) and guavin B (sr. 9, *m*/*z* 676) along with the geraniin isomers (sr. 10, *m*/*z* 953 and sr. 12, *m*/*z* 710). The peaks with molecular ion *m*/*z* ratios of 643.7 and 660.0, respectively, were identified as belonging to quercetin galloylhexoside derivatives. The fragment ion at *m*/*z* 499.3 belongs to the HHDP glucose isomer after the loss of HHDP and glucogallin compounds. The above analysis of the mass chromatograms validates the hypothesis that the *P. guajava* leaf extract is indeed enriched in the polyphenols and aromatic compounds,^61^ which can be utilized as reducing agents as well as acting as the stabilizing medium for metal oxide nanostructures.

**Figure 2:**
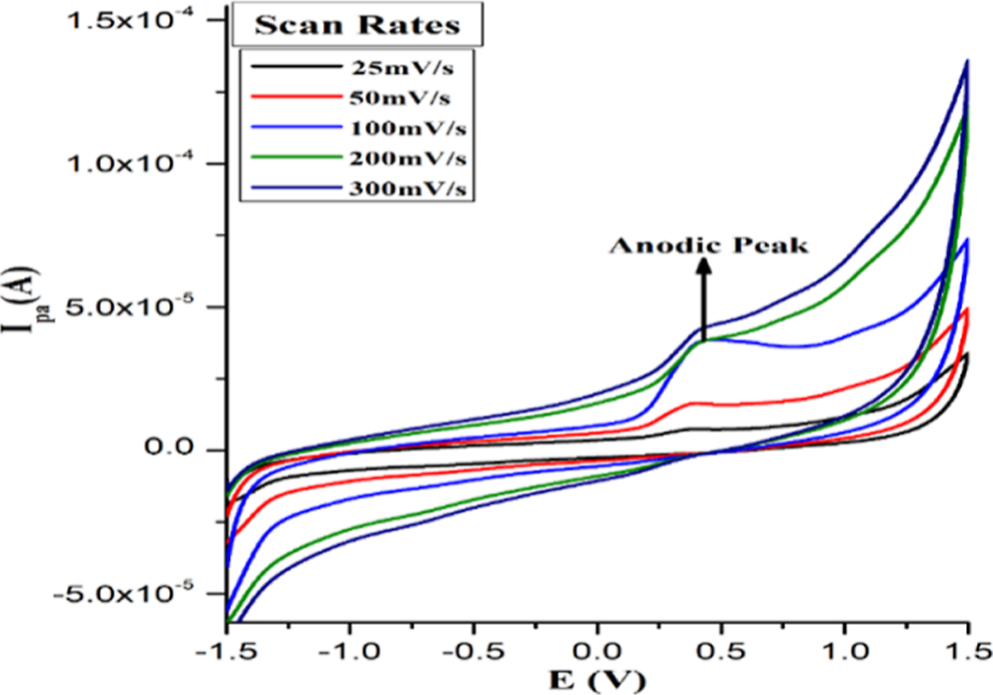
Cyclic voltammograms of the *P. guajava* leaf extract at different scan rates.

Cyclic voltammetry (CV) studies were carried out to determine the redox potential of major phytochemical components in the *P. guajava* leaf extract. Figure 2 depicts cyclic voltammograms of the green extract at different scan rates, ranging from 25 to 300 mV/s. The immediate observation is the presence of an anodic peak at 0.46 V, which is associated with oxidation centers present in polyphenols that quickly oxidize to yield a phenoxonium ion. The absence of a cathodic peak indicates that the oxidation is followed by a chemical reaction which rapidly utilizes the generated phenoxonium ion through an electron chemical (EC) mechanism, coupling, or nucleophilic attack.^62^ An increase in the scan rate (up to 300 mV/s) leads to a proportional increase in the anodic peak current, suggesting an electron transfer mechanism.^63,64^ An anodic peak current (*I*_pa_ = 38.50 μA) at an oxidation potential (*E*_oxi_ = 0.45 V) was obtained at a scan rate of 100 mV. A linear increase in anodic peak current is observed with increasing scan rate (Figure S2), indicating a diffusion-controlled reaction according to the Randles–Sevcik equation.^65^ The CV studies of the green extract thereby provide a picture of the reaction mechanism, wherein the polyphenols are oxidized themselves to yield the phenoxonium ion while acting as reducing agents in the synthesis of nanostructures. The other minor biomolecules are left to act as stabilizing agents during the reaction, thereby controlling the size of nanostructures.

The mass chromatographs indicate the major entities present in the *P. guajava* extract in large amounts (Figure S1) that may prevent agglomeration of the synthesized nanostructures by acting as capping agents, thereby also acting as size-controllers. Table 1 shows the identified biomolecules with their retention times, and a careful observation points toward the presence of polyphenols and flavonoids. For example, the compound tetrahydroxystilbene galloyl hexoside (sr. 5) exhibits a molecular ion peak at *m*/*z* 556, while the fragment ion at *m*/*z* 380 (sr. 1) is attributed to tetrahydroxystilbene galloyl after the loss of a hexosyl residue. Similarly, the guava-coumaric acid isomer derivative (sr. 6, *m*/*z* 622.5) and its fragment ion (sr. 2, *m*/*z* 423) after the loss of *p*-coumaroyl or feruloyl residues can clearly be identified. Additionally, there is evidence of the presence of glycoside (sr. 4, *m*/*z* 535) and guavin B (sr. 9, *m*/*z* 676) along with the geraniin isomers (sr. 10, *m*/*z* 953 and sr. 12, *m*/*z* 710). The peaks with molecular ion *m*/*z* ratios of 643.7 and 660.0 were identified as belonging to quercetin galloylhexoside derivatives. The fragment ion at *m*/*z* 499.3 belongs to the HHDP glucose isomer after the loss of HHDP and glucogallin compounds. The above analysis of the mass chromatograms validates the hypothesis that the *P. guajava* leaf extract is indeed enriched in the polyphenols and aromatic compounds,^61^ which can be utilized as reducing agents as well as acting as the stabilizing medium for metal oxide nanostructures.

CV studies were carried out to determine the redox potential of major phytochemical components in the *P. guajava* leaf extract. Figure 2 depicts cyclic voltammograms of the green extract at different scan rates, ranging from 25 to 300 mV/s. The immediate observation is the presence of an anodic peak at 0.46 V, which is associated with oxidation centers present in polyphenols that quickly oxidize to yield a phenoxonium ion. The absence of a cathodic peak indicates that the oxidation is followed by a chemical reaction which rapidly utilizes the generated phenoxonium ion through an EC mechanism, coupling, or nucleophilic attack.^62^ An increase in the scan rate (up to 300 mV/s) leads to a proportional increase in the anodic peak current, suggesting an electron transfer mechanism.^63,64^ An anodic peak current (*I*_pa_ = 38.50 μA) at an oxidation potential (*E*_oxi_ = 0.45 V) was obtained at a scan rate of 100 mV. A linear increase in anodic peak current is observed with increasing scan rate (Figure S2), indicating a diffusion-controlled reaction according to the Randles–Sevcik equation.^65^ The CV studies of the green extract thereby provide a picture of the reaction mechanism, wherein the polyphenols are oxidized themselves to yield the phenoxonium ion while acting as reducing agents in the synthesis of nanostructures. The other minor biomolecules are left to act as stabilizing agents during the reaction, thereby controlling the size of nanostructures.

### 2.2 X-ray Diffraction Analysis

**Figure 3:**
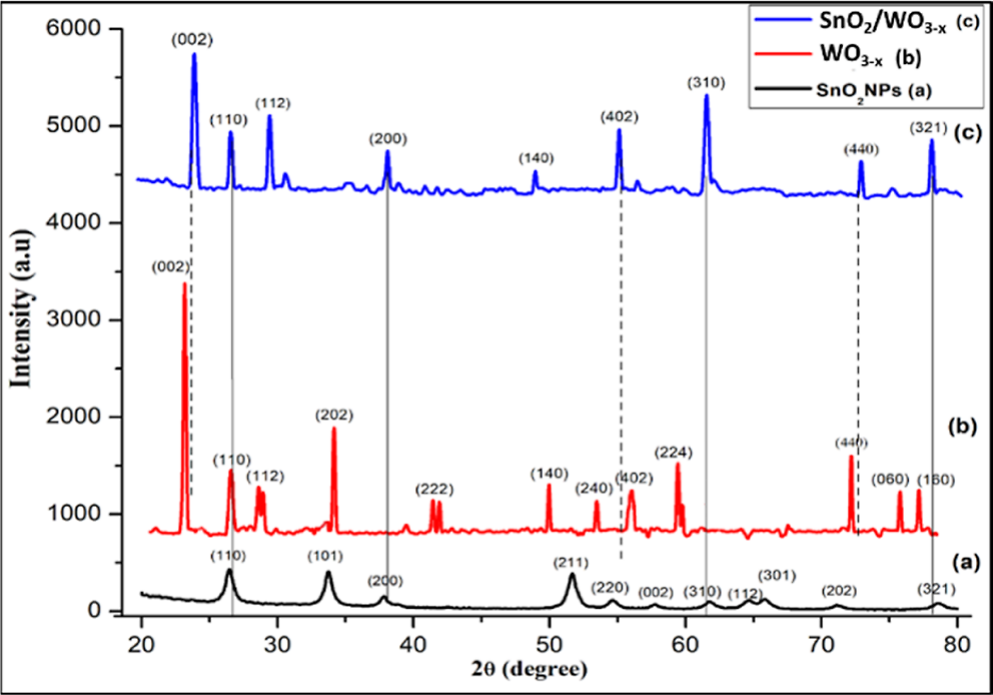
XRD patterns of (a) SnO_2_, (b) WO_3_, (c) SnO_2_/WO_3–*x*_ monometallic and hetero-oxide nanostructures in black, red, and blue colors, respectively.

The X-ray diffraction (XRD) patterns of the as-synthesized monometallic (SnO_2_ and WO_3_) and hetero-oxide (SnO_2_/WO_3–*x*_) nanostructures are shown in Figure 3. The trace pattern (a) shows the characteristic diffraction peaks of SnO_2_ at 26.52, 33.76, 37.50, and 78.52° corresponding to the (110), (101), (200), and (321) planes, respectively. An average crystallite size of ∼22 nm was calculated for SnO_2_ nanoparticles from the Scherrer equation.^66^ SnO_2_ nanocrystals (JCPDS file no. 41-1445) were found to have a tetragonal rutile structure based on the obtained lattice parameters (*a* = *b* = 4.739 Å and *c* = 3.186 Å). Trace b depicts the XRD pattern of WO_3_ nanostructures with six prominent diffraction peaks at 2θ = 23.12, 28.50, 34.156, 49.95, 56.11, and 72.16° corresponding to indexed planes (002), (112), (202), (140), (402), and (440), respectively. The highly crystalline and WO_3_ nanostructures give an average crystallite size of ∼34 nm with a monoclinic crystal lattice (*a* = 7.297 Å, *b* = 7.539 Å, and *c* = 7.688 Å) (JCPDS file no. 43-1035).

The powder XRD pattern of non-stoichiometric SnO_2_/WO_3–*x*_ nanostructures in a 2:1 ratio (Figure 3, trace c) shows narrow diffraction peaks assigned to the (002), (110), (112), (200), (140), (402), (310), (440), and (321) planes of both SnO_2_ and WO_3_ monometallic oxide nanostructures. Slight peak shifts in 2θ values, as indicated by dotted lines, clearly infer that the synthesized material is composed of the SnO_2_/WO_3–*x*_ bimetallic single phase.^67^ Moreover, the non-stoichiometric SnO_2_/WO_3–*x*_ nanostructures having a ratio of 2:1 exhibited the diffraction peaks which match quite well with the peaks observed in the case of pure SnO_2_ and WO_3_ nanostructures, respectively. From the Scherrer equation, the average crystallite size of the SnO_2_/WO_3–*x*_ nanostructures was found to be ∼34 nm. Notably, few diffractions peaks of the WO_3_ nanostructure are missing in the XRD pattern of non-stoichiometric SnO_2_/WO_3–*x*_ nanostructures (blue line Figure 3). For example, the peaks at 202 and 222 *hkl* planes which are present in the XRD pattern of pure WO_3_ (red line, Figure 3) are not there in the heterostructure, which is possibly due to the mixed oxidation state of tungsten.^68^

### 2.3 FT-IR Spectral Analysis

**Figure 4:**
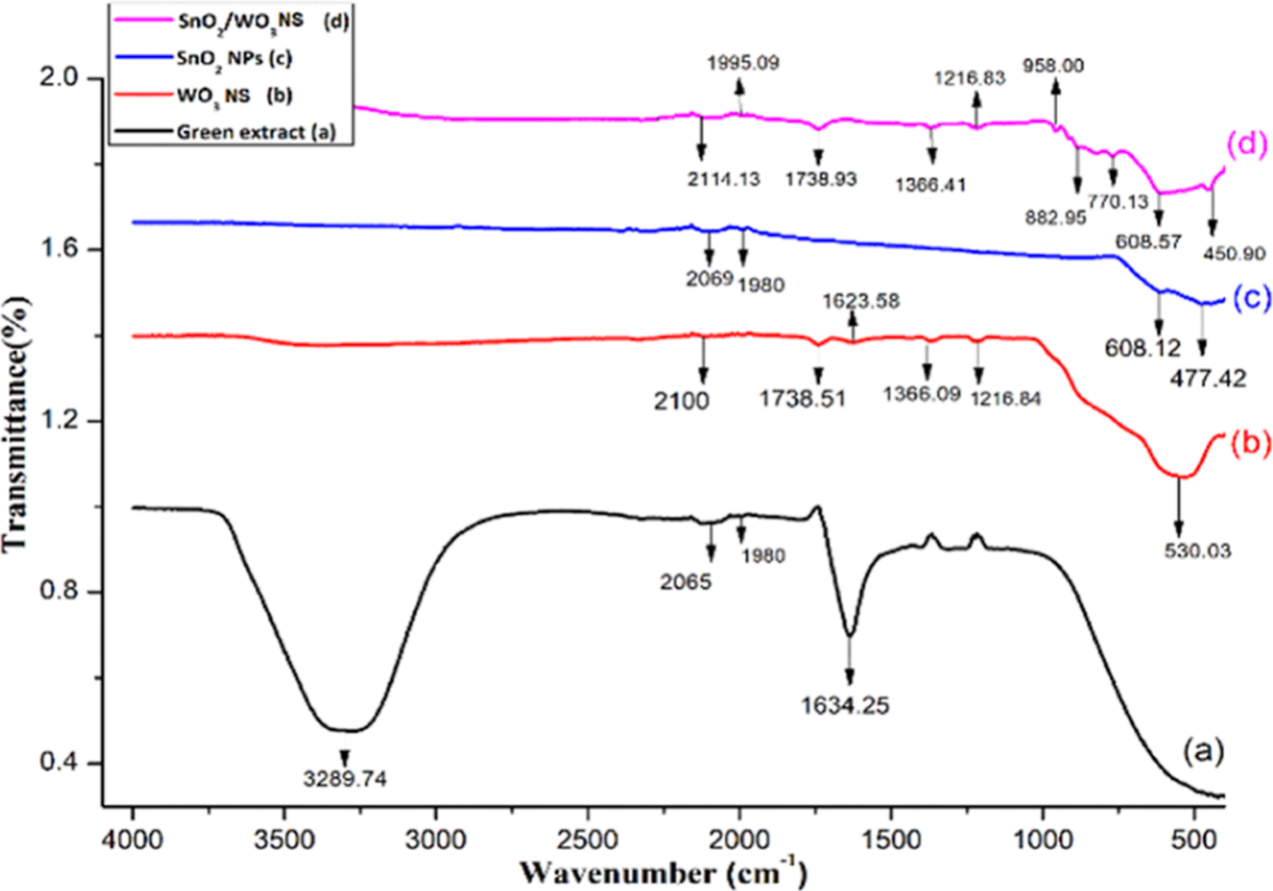
FT-IR spectra of the (a) *P. guajava* leaf extract, (b) WO_3_ NSs, (c) SnO_2_ NPs, and (d) SnO_2_/WO_3–*x*_ nanostructures.

The Fourier transform infrared (FT-IR) spectra of the pure green extract and the monometallic/bimetallic nanostructures are shown in Figure 4 to identify the functional groups acting as reducing and stabilizing agents during the synthesis stage. The broad band in (a) (green extract) centered at 3289 cm^–1^ can be attributed to the −OH stretching vibrations, while the smaller bands appearing at 2065 and 1980 cm^–1^ arise due to respective −C≡C stretches and −C-H bends, indicating the presence of alkyne and aromatic compounds in the plant extract.^69^ Sharp bands at 1760 and 1634 cm^–1^ correspond to −C=C stretching and −OH group bending vibration, respectively, indicating the alkenes and aromatic moieties in flavonoids and phenolic compounds. A characteristic band in the FTIR spectrum (Figure 4b) for WO_3_ nanostructures at 530 cm^–1^ was observed due to W–O–W stretching vibrations. In the case of SnO_2_ NPs seen in Figure 4c, characteristics bands appear at 608 and 477 cm^–1^ corresponding to the Sn–O–Sn anti-symmetric and Sn–O terminal stretching vibration modes, respectively. The presence of the characteristic peaks of both the metal oxides in Figure 4d for SnO_2_/WO_3–*x*_ nanostructures is documented with slight shifts, confirming the idea that the metal oxide nanostructures retain their optical characteristics in the bimetallic assembly. Absorption bands for Sn–O–Sn anti-symmetric and Sn–O terminal stretching vibrations occur at 608 and 450 cm^–1^, respectively, while bands at 958 and 770.13 cm^–1^ refer to ν(W–O) and γ(W–O–W) stretching modes.^70^

It is already established from the LC–MS analysis of the *P. guajava* leaf extract that it is enriched with guavin B, HHDP glucose isomers, and quercetin derivatives which undergo hydrolysis in aqueous media to produce different organic acids and catechins where the produced electrons contribute toward the reduction to nanostructures. The remaining charged functional moieties present in the extract serve as the stabilizing agent by efficiently interacting with the surface of the engineered nanostructures,^71^ which causes slight shifts in the absorption bands. For instance, trace 4b confirms the existence of the phyto-molecules as the characteristic bands of the plant extracts were observed at 2100, 1738, and 1366 cm^–1^ with slight or no change in comparison to the spectra of the pure extract. The presence of these bands clearly indicates that phenolic compounds have strong ability to bind with metal oxide NSs, thus preventing their agglomeration.^72^ Similar trends were observed in the case of SnO_2_ and SnO_2_/WO_3–*x*_ nanostructures.

### 2.4 UV–Vis Studies of Monometallic Oxide and Hetero-oxide Nanostructures

**Figure 5:**
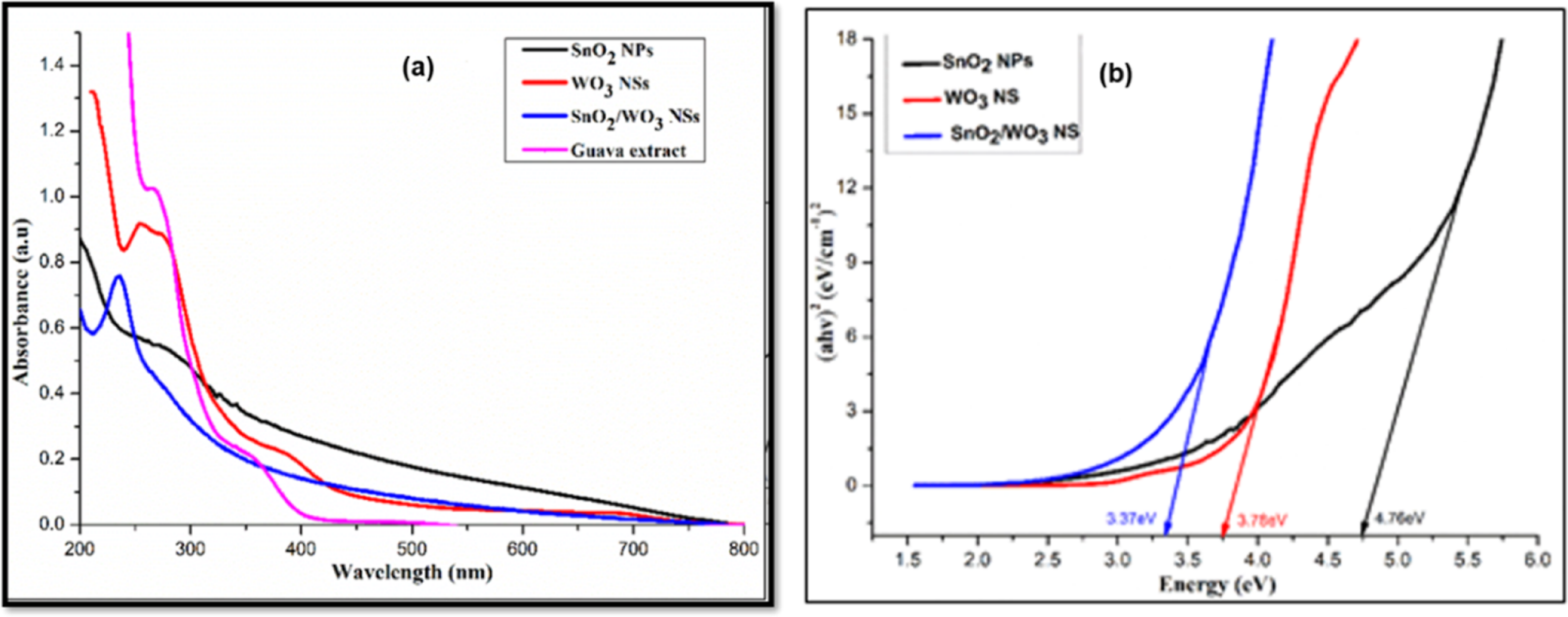
(a) UV–visible spectra of (black) SnO_2_ NPs, (red) WO_3_ NSs, (blue) SnO_2_/WO_3–*x*_ nanostructures, and (magenta) *P. guajava* leaf extract and (b) optical band gaps of (black) SnO_2_ NPs, (red) WO_3_ NSs, and (blue) SnO_2_/WO_3–*x*_ bimetallic oxide NSs obtained from Tauc plots.

The *P. guajava* green extract and the synthesized nanostructures were characterized by UV–visible spectroscopy, as depicted in Figure 5. The spectra of nanostructures show prominent absorption peaks arising out of surface plasmonic resonance (SPR), attributed to the synchronized oscillation of electrons on the surface of the nanostructures.^73,74^ Trace (a) (black) depicts the characteristic SPR peak of SnO_2_ NPs at 285 nm corresponding to previously reported findings.^75^ It has been shown that the position, width, and intensity of the SPR bands can be directly correlated to the size, homogeneous distribution, and concentration of the nanoparticles.^76^ The WO_3_ nanostructures exhibit an SPR band at 260 nm under ambient conditions.^77,78^ Moreover, some low intensity peaks at 265 and 389 nm in the UV spectrum of the green extract (trace (d) (magenta)) can be attributed to active polyphenolic components.^79^ The intensity and position of these peaks in the UV–visible spectrum of WO_3_ nanostructures show slight shifts, suggesting the involvement of polyphenols in the green extract in the capping of synthesized NSs.^80^

The characteristic SPR bands in the UV–vis spectra of the nanostructures can also provide useful information regarding their monometallic or bimetallic nature. The appearance of a single SPR band in the UV–visible spectrum of SnO_2_/WO_3–*x*_ nanostructures (trace (a)) at 235 nm indicates the presence of synergistic effects in generating single-phase isomorphic SnO_2_/WO_3–*x*_ nanostructures. These synergistic aspects are also confirmed by the shift in SPR peaks in hetero-oxide nanostructures compared to those observed for monometallic oxides.^81,82^ Tauc plots generated from the UV–visible spectroscopic data (Figure 5b) are used to calculate the optical band gap of nanostructures (*E*_g_ in eV) using the empirical formula
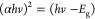
1where *hv* represents the photon energy and α is the absorptivity co-efficient of the nanomaterial. The optical band gaps for SnO_2_ NPs, WO_3_ NSs, and SnO_2_/WO_3–*x*_ nanostructures were calculated to be 4.76, 3.78, and 3.37 eV, respectively, as shown in trace (b), providing essential information about the photocatalytic potential of the engineered assemblies. We observed that there is a substantial decrease in the band gap in bimetallic oxide nanostructures compared to their monometallic counterparts, indicating their enhanced potential in photocatalysis. Notably, in this case, the high band gap of WO_3_ can be possibly attributed to the presence of a mixture of +5 and +6 oxidation states of tungsten, as reported in the literature and may also be indicated by the presence of extra peaks in the diffraction pattern of WO_3_ (Figure 3).^68^

### 2.5 Morphology and Compositional Analysis of Nanostructures

**Figure 6:**
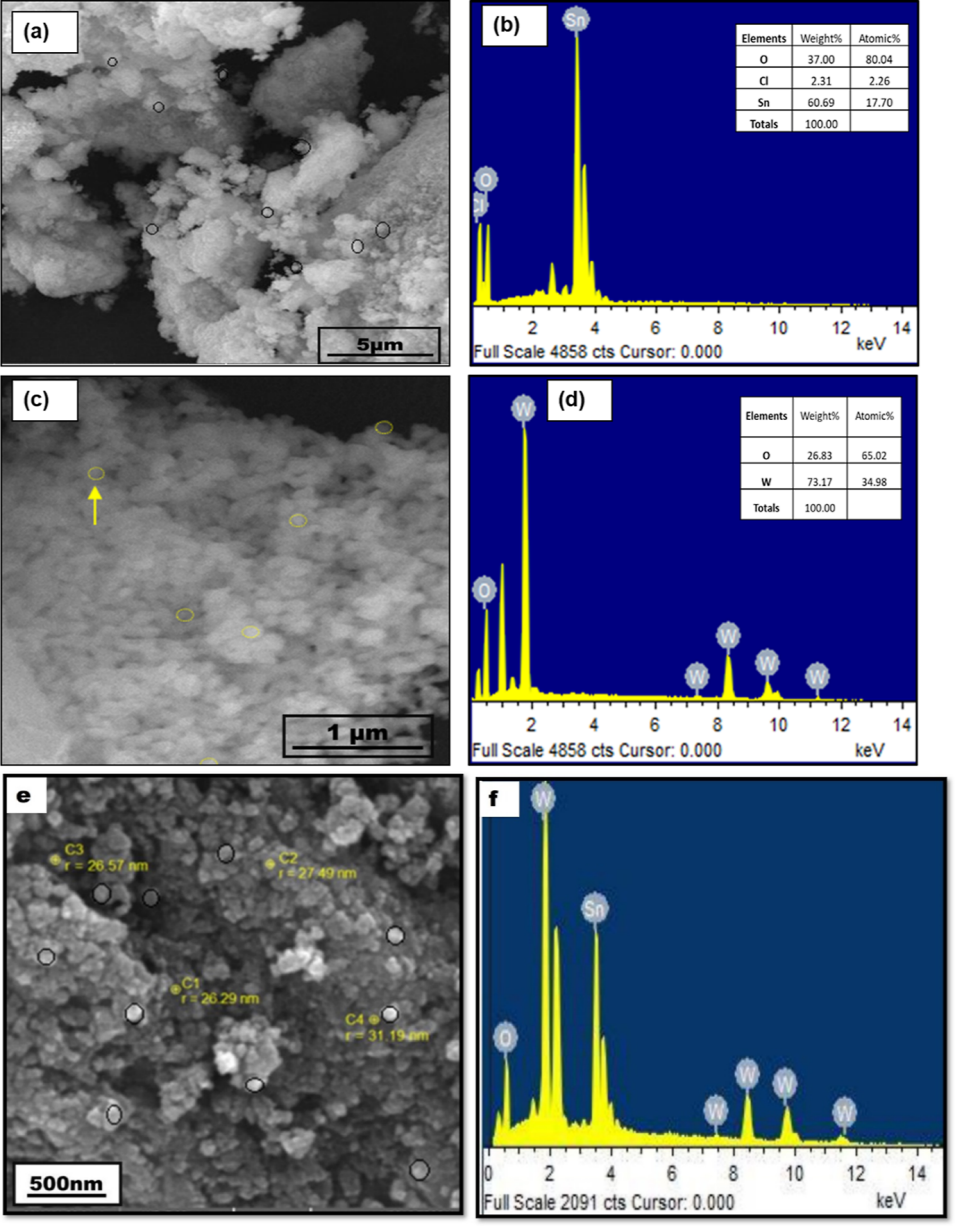
(a) SEM micrographs of SnO_2_ NPs, (b) EDS analysis of SnO_2_ NPs, (c) SEM images of WO_3_ NSs, (d) EDS spectrum of WO_3_ NSs, (e) SEM image of SnO_2_/WO_3–*x*_ NSs, and (f) EDS spectrum of SnO_2_/WO_3–*x*_ NSs.

The shape and size of SnO_2_ NPs, WO_3_ monometallic oxide, and SnO_2_/WO_3–*x*_ nanostructures were studied by scanning electron microscopy (SEM) and transmission electron microscopy (TEM). The SEM micrographs and elemental composition of the SnO_2_ and WO_3_ monometallic nanostructures are shown in Figure 6. SnO_2_ NPs prepared from a green route are observed to have a spherical shape with agglomerates (Figure 6a). The characteristic peaks of stannum (Sn; 60.69%) and oxygen (O; 37.00%) are indicated in the energy-dispersive spectrum (Figure 6b). On the other hand, the SEM micrographs of WO_3_ NSs (Figure 6c), demonstrate a quasi-spherical shape with slight agglomeration; the energy-dispersive spectrometry (EDS) spectrum (Figure 6d) reveals the existence of tungsten and oxygen in a weight percentage of 73.17 and 26.83%, respectively. The size and morphological features of the engineered bimetallic oxide nanostructures are provided in Figure S3a, demonstrating uniform spherical shaped nanostructures with a size range of 26–32 nm. The aggregation is attributed to the difference in capping ability of various naturally derived compounds present in the green extract. The EDS spectrum provided in Figure S3b shows the presence of tungsten (W; 34.45%), stannum (Sn; 32.28%), and oxygen (O; 33.27%), indicating a high level of purity.

**Figure 7:**
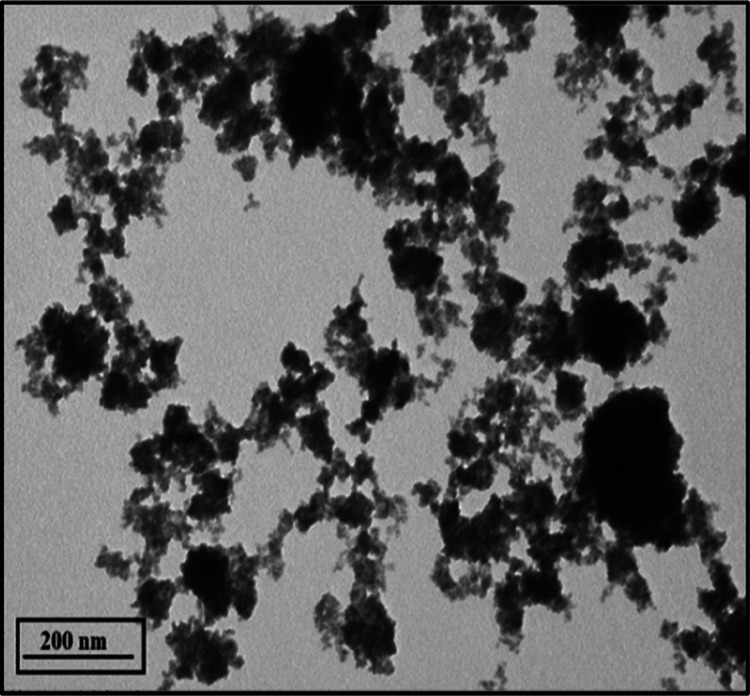
TEM micrograph of SnO_2_/WO_3–*x*_ nanostructures (scale bar: 200 nm).

The TEM micrographs of the synthesized bimetallic oxide nanostructures are shown in Figure 7. The engineered nanostructures are observed to have a nearly quasi-spherical shape on a scale of 200 nm. The micrographs reveal interconnected SnO_2_/WO_3–*x*_ nanostructures with much less aggregation compared to their monometallic oxide counterparts.

### 2.6 Photodegradation of MB Dye through Synthesized Nanostructures

**Figure 8:**
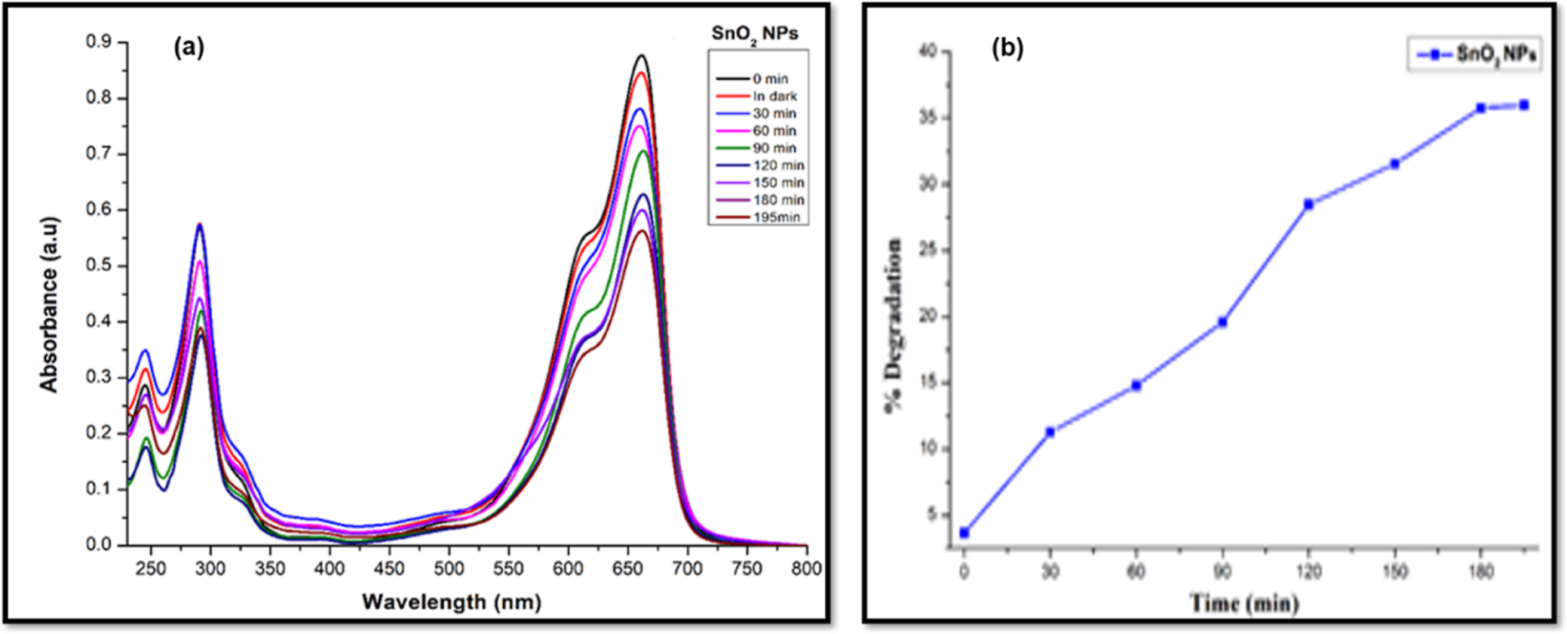
(a) UV–vis spectra of MB dye with and without monometallic SnO_2_ NPs at regular time intervals and (b) plot of percentage degradation of MB dye as a function of irradiation time.

The monometallic and hetero-metallic oxide nanostructures were investigated as potential photocatalysts for the degradation of MB dye under ambient conditions and UV illumination. In photocatalysis, the light-induced redox reactions come into play as electron–hole pairs are produced on the surface of the metal oxide nanostructures; tuning the band gap of the nanostructures can lead to effective reduction/oxidation of organic dye molecules on UV irradiation. Computational studies on the MB dye were performed in the Gaussian. Inc (USA) software package using the B3LYP level of theory with a 6-311G basis set to obtain the optical band gap of the dye.^83^ The frontier molecular orbitals were computed to have energies of −6.292 eV (LUMO) and −8.783 eV (HOMO), with a band gap of 2.491 eV. The photocatalytic performance of the as-synthesized monometallic SnO_2_ and WO_3_ nanostructures was evaluated by photo-degradation of MB dye in aqueous solution under UV light irradiation (Figure 8). MB shows two strong absorption bands at 662 and 290 nm. However, on the addition of SnO_2_ NPs under UV irradiation, the absorption spectra of MB show a decrease in absorbance at regular intervals of time, as shown in Figure 8a. The percentage of degradation increases as a function of irradiation time; it was observed that monometallic SnO_2_ NPs degraded 35.7% of the MB dye within 195 min of irradiation. The percentage degradation of MB dye versus time was recorded in Figure 8b. The rate constant of dye degradation (*k*) and regression coefficient (*R*^2^) were calculated to be 2.2 × 10^–3^ min^–1^ and 0.9855, respectively, from the pseudo-first-order rate law equation^84^ (Figure S4a).

**Figure 9:**
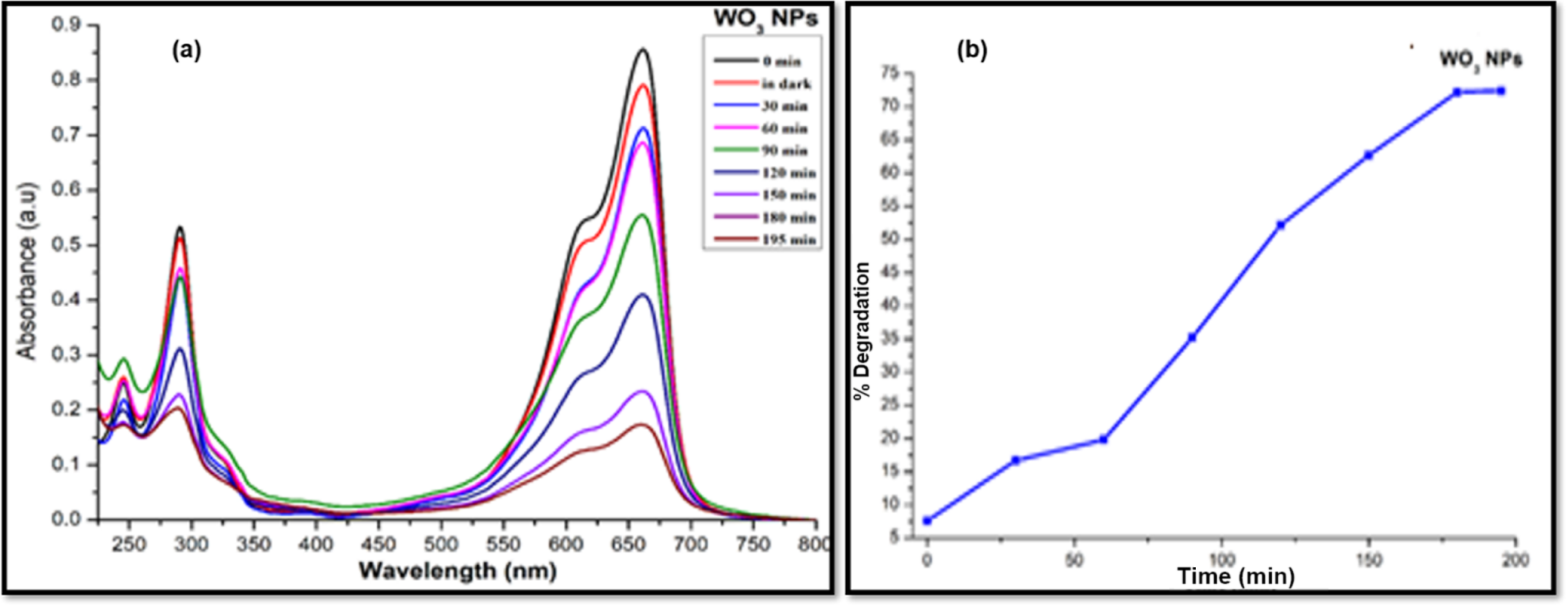
(a) UV–vis spectra of photocatalytic degradation of MB dye by monometallic WO_3_ NSs and (b) percentage degradation of MB vs irradiation time.

Figure 9a,b shows the absorption spectra of MB dye on the addition of WO_3_ monometallic NSs, percentage degradation of the dye as a function of irradiation time, and the reaction kinetics. In the case of monometallic WO_3_ NSs, it was observed that on the addition of the photocatalyst to aqueous solution of MB dye and UV irradiation, almost 74.5% of the dye degraded within 195 min of irradiation, which is a much improved performance compared to monometallic SnO_2_ NPs. The rate constant for dye degradation (*k*) and regression coefficient (*R*^2^) were calculated from pseudo-first-order rate law equation as 8.6 × 10^–3^ min^–1^ and 0.938, respectively (Figure S4b).

**Figure 10:**
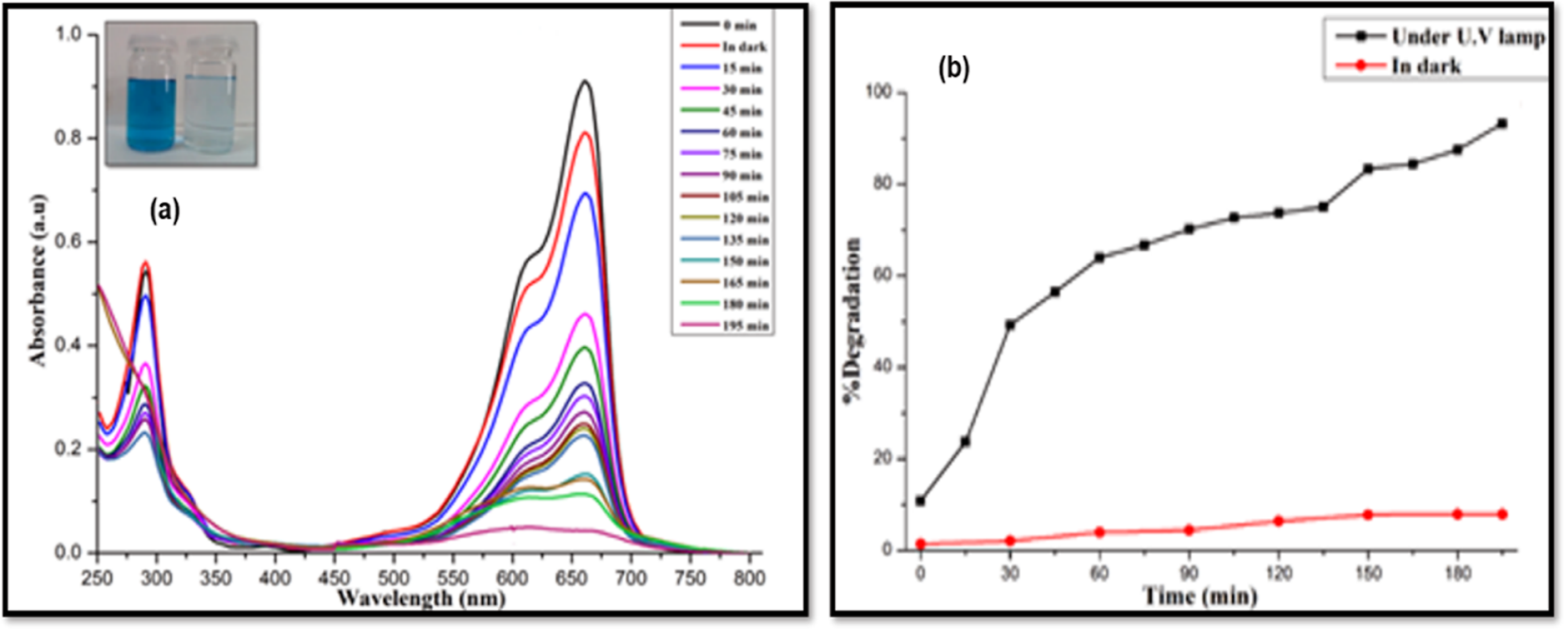
(a) UV–vis spectra of photocatalytic degradation of MB dye by hetero-SnO_2_/WO_3–*x*_ NS and (b) percentage degradation of MB vs irradiation time.

It has previously been noted from the Tauc plots that the band gap of SnO_2_/WO_3–*x*_ nanostructures (3.37 eV) is much lower than those for SnO_2_ (4.76 eV) and WO_3_ (3.78 eV) NSs. The enhanced photocatalytic activity of the bimetallic SnO_2_/WO_3–*x*_ is apparent from their absorption spectra (Figure 10a), which show a 93.52% photochemical degradation of the MB dye within 195 min, under UV irradiation. The percentage degradation was measured by plotting the decrease in dye concentration [(*A*_o_ – *A*)/*A*] as a function of time, as shown in Figure 10b. The photodegradation of MB with bimetallic SnO_2_/WO_3_ nanostructures follows the pseudo-first-order rate law given in eq 2. The rate constant for dye degradation (*k*) and regression coefficient (*R*^2^) are calculated from Figure S4c to be 9.77 × 10^–3^ min^–1^ and 0.9774, respectively.
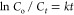
2

It is surmised that the decreased band gap of the bimetallic SnO_2_/WO_3–*x*_ photocatalysts leads to the increase in photon-harvesting and photo-responsive capability^85^ as well as enhanced charge carrier separation in the system and inhibits the recombination rate of the photogenerated electron–hole pair.^86^ This can also be attributed to the fact that the bimetallic oxide nanostructures are composed of two different metal oxides combined in a single phase due to the synergistic effect and offer increased specific surface area for photogenerated electron–hole pairs. The synergistic electronic effect means that the electrons can readily transfer from SnO_2_ to WO_3_ within the non-stoichiometric hetero-oxide nanostructure, leading to an increase in the electron density on the surface, thereby improving its photocatalytic activity.^87^ For further assurance, the dye solutions were treated with bimetallic SnO_2_/WO_3–*x*_ in the dark under similar conditions; only a 7–8% removal of MB dye was observed due to adsorption of dye on the surface of the photocatalyst. The aqueous dye solutions were irradiated with UV light in the absence of the photocatalyst with only 5–6% removal, confirming that the degradation under UV light is greatly assisted in the presence of the photocatalyst, in particular, the bimetallic SnO_2_/WO_3–*x*_ NS.^88^

### 2.7 Effect of pH on Photodegradation

**Figure 11:**
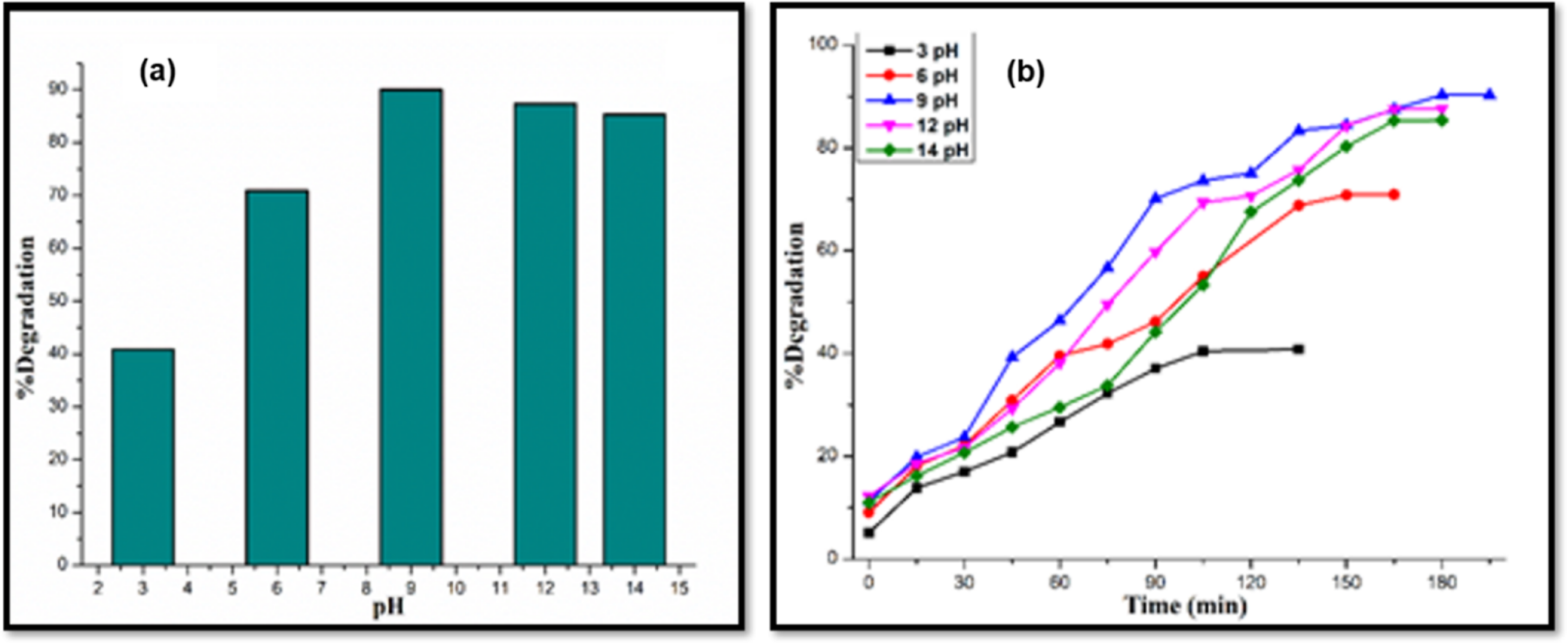
(a) Percentage degradation of MB dye at different pH values and (b) percentage degradation of MB against irradiation time at different pH values.

The effect of pH on photodegradation is dependent on the charges on the dye, surface charges on the photocatalyst,^89^ and the degradation mechanism, namely hydroxyl radical attack, direct oxidation or reduction by photogenerated holes or electrons in the conduction band.^90^ A series of experiments were conducted to analyze the role of pH variation (from 3 to 14) using 0.1 M NaOH and 0.01 M HCl solutions. The concentrations of the MB dye and the SnO_2_/WO_3_ photocatalyst were kept constant at 10 ppm and 15 mg, respectively. It was observed that upon increasing the pH from 3 to 9, the percentage degradation of MB dye under UV light increases, with a maximum 90% degradation observed at pH 9. This could be due to the cationic nature of MB^91^ with positively charged sulfur atoms in aqueous medium, which show enhanced interactions with the negatively charged photocatalyst surface in basic conditions. In alkaline medium, the hydroxyl radical acts as the primary oxidant and can easily be formed by reaction between the hydroxide ion and photogenerated hole, leading to maximum degradation.^92^ Moreover, higher pH is more favorable for sulfur-containing organic oxidation due to minimal photocatalyst corrosion.^93^ The effect of pH on percentage degradation of MB and irradiation time is depicted in Figure 11.

### 2.8 Effect of Photocatalyst Dose and Dye Concentration

Six sets of experiments were conducted to finalize the optimal photocatalyst dose for maximum dye degradation under UV light irradiation. The amount of photocatalyst was increased from 4 to 15 mg, keeping a fixed concentration of dye (10 ppm) in 100 mL of aqueous solution at pH 9. It was observed that the percentage degradation of the dye increases due to availability of a large number of active sites and hydroxyl and superoxide radicals. On further increase in the photocatalyst concentration, the percentage degradation remains constant (Figure S5) with a slight shadowing effect, wherein the turbidity due to high dose leads to hindrance in penetration depth^94^ and scattering of light^95,96^ and the agglomeration of photocatalyst leads to non-availability of its surface for photon absorption.

**Figure 12:**
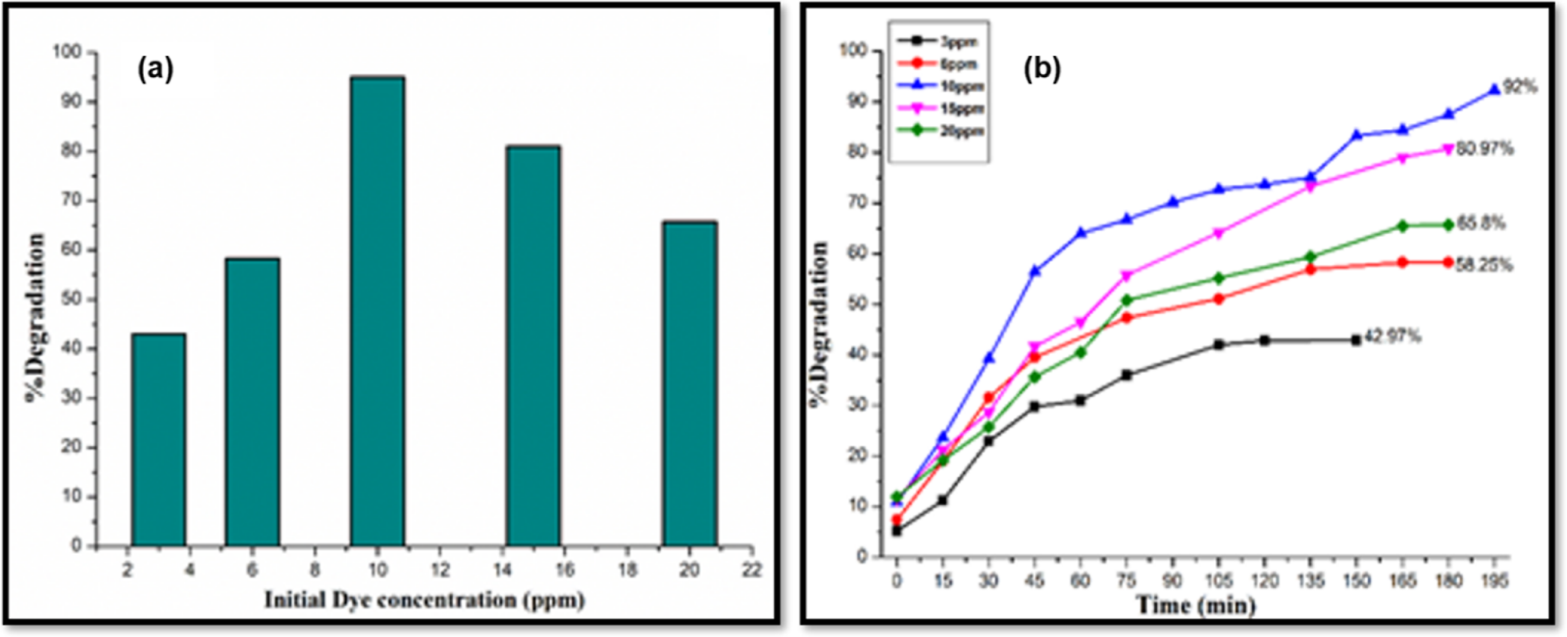
(a) Percentage degradation of MB dye at varying initial concentration and (b) % degradation of MB dye with irradiation time at different dye concentrations.

Additionally, the effect of initial concentration of MB was also studied in a range of 3–20 ppm in 100 mL of water at the optimal conditions of pH 9 and photocatalyst concentration (10 mg) (Figure 12). The best degradation efficiency (93.5%) is achieved when the initial concentration of dye is set at 10 ppm. On further increase, a considerable decrease in % degradation was observed due to unavailability of active sites on the surface of the photocatalyst, obstruction in light penetration in a concentrated dye solution, low transport to the photocatalyst surface,^97^ as well as self-inhibition.^98^

The results of degradation of MB dye were also compared under similar conditions when tap water was used to prepare aqueous solutions of dye instead of DI water. The results in Figure S6a show decreased degradation efficiency (71.5%) and low rate constant (0.00547 min^–1^) compared to those of DI water (93%). This behavior could be due to the presence of metal ions in tap water (Na^+^, Cl^–^, K^+^, Ca^2+^, Fe^3+^, etc.) and organic and inorganic molecules acting as competitive species, leading to reduced photocatalytic activity.^99^ Additionally, the stability, reusability, and durability of the photocatalyst catalyst were studied for five 5 consecutive cycles (Figure S6b). For this purpose, the bimetallic oxide NSs were recovered by centrifugation after a degradation experiment, washed, dried, and reused for another set of photodegradation experiment, keeping all other parameters constant. Results indicate a constant % degradation (93%) up to 3 cycles. A slight decrease in the activity of the photocatalyst was observed in the next 2 cycles with % degradation down to 85.1 and 80.02%, respectively, due to the blocking of active sites of the photocatalyst by adsorbed dye molecules^100^ and wastage of the photocatalyst during the recovery process.

## 3 Experimental Section

### 3.1 Materials and Chemicals

Sodium tungstate dihydrate (NaWO_4_·2H_2_O) (≥99.0%), tin tetrachloride anhydrous (SnCl_4_) (99.99%), and MB dye (>95%) were purchased from Sigma-Aldrich. *P. guajava* (Guava) leaves were collected from the botanical gardens of Quaid-i-Azam University, Islamabad, Pakistan. All aqueous solutions were prepared in DI water. All the utilized chemical reagents were of analytical grade and used directly without any further purification. The glassware was thoroughly cleaned by using freshly prepared aqua regia followed by washing through chromic acid. Finally, it was rinsed with DI water.

### 3.2 Preparation of the *P. guajava* Leaf Extract

The extract from *P. guajava* leaves was prepared using DI water as the extracting solvent.^101^ Water is preferred over other solvents, such as methanol or ethanol, due to its low molecular weight, high boiling point, and purity, as the phenolics which act as reducing and capping agents are obtained in high yield in water. Collected and cleaned *P. guajava* leaves were dried in direct sunlight for 3–4 days and grounded. The extract was prepared in 400 mL of DI water using 30 g of dried leaves under constant stirring at 65 °C for 4 h. A dark brown extract was obtained after filtration which was stored at 4 °C until further use.

### 3.3 Green Synthesis of SnO_2_ Nanoparticles

1.5 M SnCl_4_ aqueous solution (40 mL) was added dropwise to the *P. guajava* (guava) leaf extract in a 1:1 ratio (v/v %). The reaction mixture was stirred for 4 h at 60 °C after which a yellow-colored sol was obtained. After filtration and subsequent washing cycles with DI water and absolute ethanol, the yellow precipitates were dried in an atmospheric oven for 1 h at 80 °C. The bright yellow precipitates were subjected to calcination in a furnace at 400 °C for 4 h to obtain SnO_2_ nanoparticles. The dark gray colored nanoparticles of SnO_2_ obtained after calcination were stored in a glass vial for further characterization.

### 3.4 Green Synthesis of WO_3–*x*_ Nanostructures

1 M Na_2_WO_4_·2H_2_O aqueous solution (25 mL) was obtained by continuous stirring. The transparent aqueous solution was added dropwise to the *P. guajava* (guava) leaf extract in a 1:2 volumetric ratio and stirred for 4.5 h at 70 °C. After 4.5 h, reddish brown precipitates were obtained, which were filtered, washed several times with absolute ethanol and DI water, and then dried in an oven for 1 h. The precipitates were calcined at 400 °C for 6 h to obtain black colored WO_3–*x*_ nanostructures.^68^

### 3.5 In Situ Green Synthesis of Hetero-oxide SnO_2_/WO_3–*x*_ Nanostructures

A new one-pot in situ green synthesis method to obtain SnO_2_/WO_3–*x*_ nanostructures using the *P. guajava* (guava) leaf extract was developed. Aqueous solutions of SnCl_4_ (1 M) and Na_2_WO_4_·2H_2_O (2 M) were added dropwise into the green extract (100 mL) simultaneously and stirred for 5 h at 80 °C. The yellowish-brown precipitates obtained after 5 h were filtered and washed several times with DI water and absolute ethanol to remove impurities and dried in an oven for 3 h. The dried precipitates were calcined for 4 h at 600 °C to obtain light gray colored hetero-oxide SnO_2_/WO_3–*x*_ nanostructures with a much higher yield (3.2 g) compared to monometallic oxide nanostructures. The synthesized SnO_2_/WO_3–*x*_ nanostructures were stored in a glass vial for further use.

### 3.6 Characterization

The crystal structure and crystallite size of synthesized nanostructures were determined by powder XRD using a PANalytical Xpert PRO diffractometer using Cu Kα radiation with a scanning rate of 2°/min and 2θ range from 20 to 80° at room temperature. The absorption spectra of the *P. guajava* green extract and monometallic and bimetallic nanostructures were recorded using a PerkinElmer LAMBDA-3500 ultraviolet–visible (UV–vis) double-beam spectrophotometer over a wavelength range of 200–800 nm at room temperature. FTIR of nanostructures and guava leaf extract were recorded using an FTIR spectrometer (BRUKER, TENSOR-II, Germany) in a range of 400 to 4000 cm^–1^. Morphological studies of the bimetallic nanostructures were carried out using a transmission electron microscope (TEM) (JEOL 2010, Tokyo, Japan) at 200 kV. SEM imaging was done using a VEGA3 TESCAN instrument coupled with an energy-dispersive spectrometer for elemental analysis. CV is employed to determine the redox behavior of the green extract using a Gamry Interface 1000 instrument where a glassy carbon electrode was used as the working electrode, Pt foil was used as the counter electrode, and saturated calomel (Ag/AgCl) acted as the reference electrode. Potassium chloride (KCl) was used as the supporting electrolyte in a phosphate buffer of pH 7. All experiments were performed at room temperature at different scan rates of 25, 50, 100, 200, and 300 mV to determine the electrochemical potential of the green extract.

### 3.7 Preparation of the *P. guajava* Leaf Extract for LC–MS Analysis

In order to identify the biomolecules acting as reducing agents and capping entities, LC–MS analysis was performed. The samples for LC–MS analysis were prepared after extraction of an appropriate amount of dried *P. guajava* leaves in methanol. The reflux process was repeated three times until complete extraction was achieved. The extract was filtered and dried using a rotary evaporator, and 1 mg of the dried sample was redissolved in methanol, micro-filtered, and stored in plastic vials for LC–MS analysis.

The LC–MS analysis was performed using an LC-Agilent system (HP 1100 series), connected directly to a single quadrupole mass selective detector (model G2579A). The sample was scanned in a mass range of 100–1000 *m*/*z* in a negative ionization mode. The injected samples (5 μL) were analyzed in a temperature range of 60–350 °C, while the columns (J & W scientific) had dimensions of 30 m × 0.25 mm with a 0.5 μm pore size. The retention times and mass spectra of compounds were recorded, and identification of the compounds in the sample was carried out by comparing the mass spectra to those in the mass library and literature.

### 3.8 Photocatalytic Experimental Setup

The photocatalytic activity of synthesized monometallic and bimetallic oxide nanostructures was studied by detailed investigations of the degraded MB dye, a common water pollutant, under UV irradiation. A typical experiment involved adding bimetallic oxide SnO_2_/WO_3–*x*_ nanostructures (10 mg) in 100 mL of an aqueous solution of the MB dye (10 ppm) at pH 9. The experimental setup consisted of a covered glass reactor containing MB dye and the photocatalyst in aqueous medium, which is irradiated by a Philips 10 W UV lamp (30 mW/cm^2^). Prior to illumination, the suspension containing the dye and photocatalyst was stirred for 30 min to ensure adsorption equilibrium of the dye on the surface of bimetallic oxide nanostructures. The sample was illuminated afterward, and the progress of dye degradation was determined by withdrawing 2 mL of the reaction mixture at regular time intervals. After centrifugation at 3500 rpm for 5 min, the supernatant was analyzed for the MB dye concentration using a UV–visible spectrophotometer (PerkinElmer LAMBDA-3500). The degradation efficiency of MB dye was calculated at λ_max_ by the following equation.
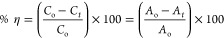
3where % η = photodegradation percentage efficiency, *A*_o_ = absorbance of original MB solution, and *A*_*t*_ = absorbance of MB dye after UV light irradiation at a certain interval of time *t* corresponding to a concentration *c*_*t*_*.*

## 4 Conclusions

In summary, SnO_2_/WO_3–*x*_ nanostructures and their monometallic counterparts were successfully prepared using a new facile green route from the *P. guajava* leave extract, followed by sintering. The one-pot green synthesis produces high yields for hetero-oxide NSs (3.2 g) compared to monometallic oxide SnO_2_ (2.1 g) and WO_3_ (0.3 g) NSs, due to synergistic nucleation. The LC–MS analysis of the *P. guajava* leaf extract provides a comprehensive map about the biomolecules present and responsible for the reduction and capping of the nanostructures, confirmed by CV. XRD analysis confirms the synthesis of highly crystalline monometallic SnO_2_ and WO_3_ and bimetallic SnO_2_/WO_3–*x*_ nanostructures with average crystallite sizes of 22, 34, and 35 nm, respectively. The FE-SEM and TEM imaging reveal the formation of clusters of spherical nanostructures with a high degree of purity. The Tauc plots from the UV–visible spectral data confirm a smaller optical band gap (3.37 eV) for SnO_2_/WO_3–*x*_ compared to SnO_2_ NPs (4.76 eV) and WO_3_ NSs (3.78 eV), indicating higher photocatalytic activity in engineered bimetallic oxides. The bio-synthesized nanostructures were used to study the photocatalytic dye degradation of MB dye under UV irradiation; the bimetallic SnO_2_/WO_3–*x*_ nanostructure exhibited an enhanced degradation efficiency of 93.5% with a rate constant (9.77 × 10^–3^ min^–1^) following pseudo-first-order kinetics. A number of parameters, that is, effect of pH, catalyst dose, and dye concentration, have been optimized for increased photocatalytic degradation efficiency of organic pollutants through SnO_2_/WO_3–*x*_ NSs with reusability up to 3 consecutive cycles.

## Supporting Information Available

LC–MS spectrum of the aqueous *P. guajava* leaf extract; plot of anodic peak current against square root of scan rate (*R*^2^ = 0.9); SEM micrograph of SnO_2_/WO_3_ bimetallic NSs; EDS spectrum of SnO_2_/WO_3_ bimetallic oxide NSs; plot of ln(*A*_o_/*A*_*t*_) against irradiation time for SnO_2_, WO_3_, and bimetallic photocatalysts; percentage degradation of MB dye (10 ppm) with increasing photocatalyst dose, degradation of MB with irradiation time at different photocatalyst doses; plot of ln(*C*_o_/*C*_*t*_) versus time in tap water; and degradation data recorded for reusability of the bimetallic oxide photocatalyst up to 5 consecutive cycles (PDF)
