# Supplementary material for: Light-Driven Catalytic Activity of Green-Synthesized SnO2/WO3–x Hetero-nanostructures
Source: ACS Omega. 2023 May 24;8(22):20042–55. doi: 10.1021/acsomega.3c02330 (PMC10249087; doi:10.1021/acsomega.3c02330)
Supplement: Supplementary file 1 — ao3c02330_si_001.pdf [file ao3c02330_si_001.pdf]

Supplementary File

Light Driven Catalytic Activity of Green Synthesized SnO_2/WO_{3-x} Hetero-Nanostructures

Faroha Liaqat*[,1], Urwa tul Vosqa[1], Fatima Khan[1], Abdul Haleem[2], Mohammed Rafi Shaik[3], Mohammed Rafiq H. Siddiqui[4], and Mujeeb Khan*[,3]

[1] Department of Chemistry, Quaid-i-Azam University, 45320, Islamabad, Pakistan

[2]CAS Key Laboratory of Soft Matter Chemistry, Department of Polymer Science and Engineering, University of Science and Technology of China, Hefei, Anhui 230026, China

[3]Department of Chemistry, College of Science, King Saud University, P.O. Box 2455, Riyadh 11451, Saudi Arabia

[4]Department of Chemistry, University of Liverpool, Liverpool L69 7ZD, United Kingdom

*Correspondence: fliaqat@qau.edu.pk (F.L.); kmujeeb@ksu.edu.sa (M.K.); Tel.: +966-11-4670439 (Muj.K.)

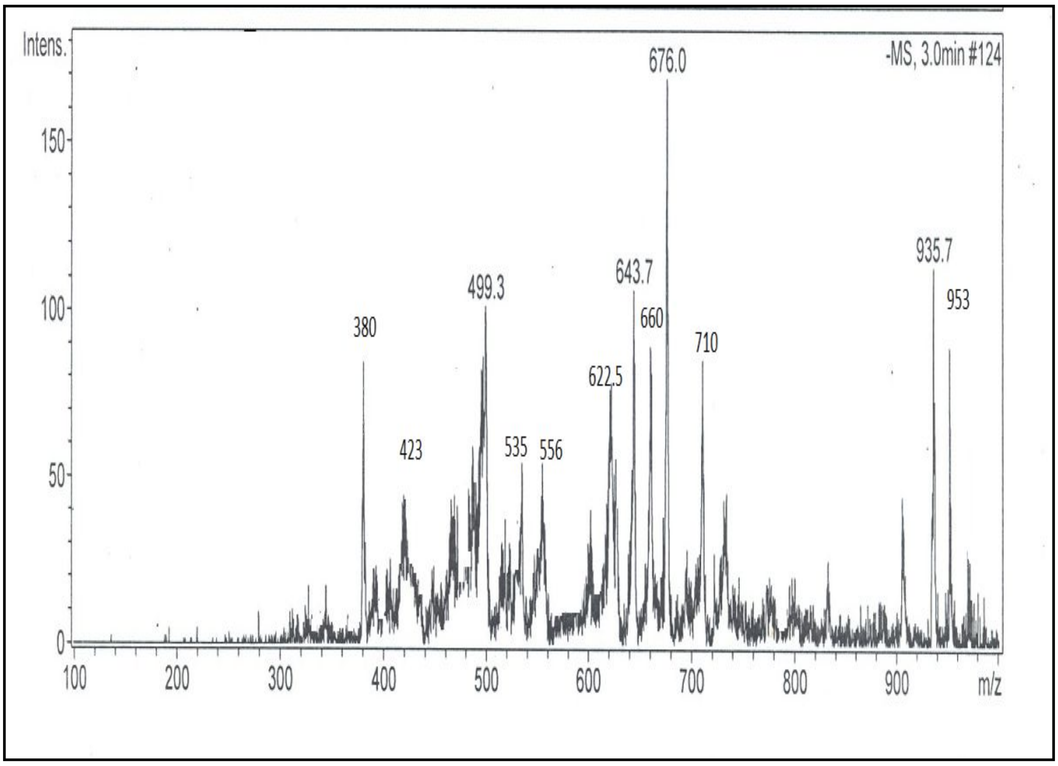

Figure S1. LC–MS spectrum of aqueous *Psidium guajava* leaves extract

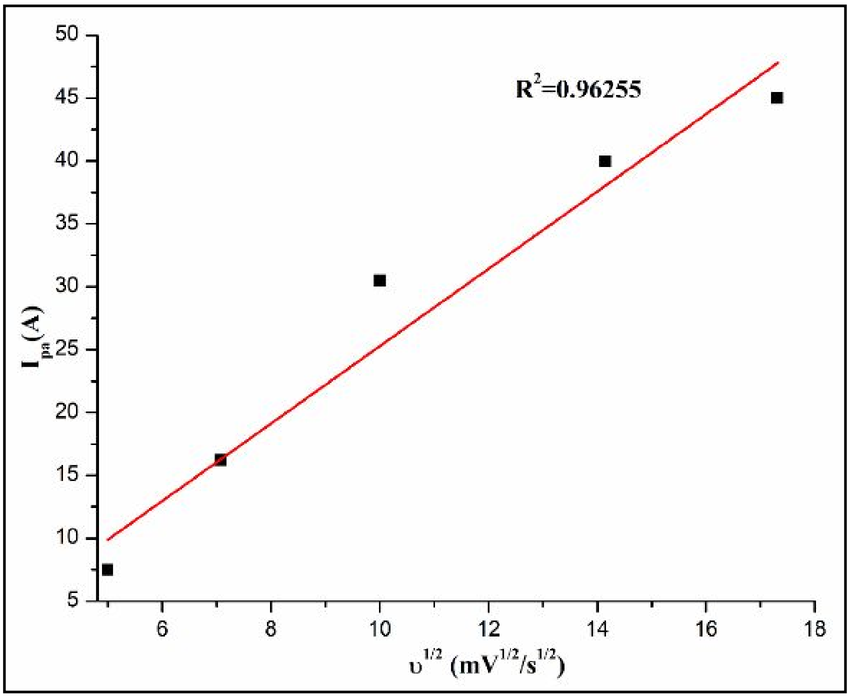

Figure S2. Plot of anodic peak current against square root of scan rate ($R^2 = 0.9$).

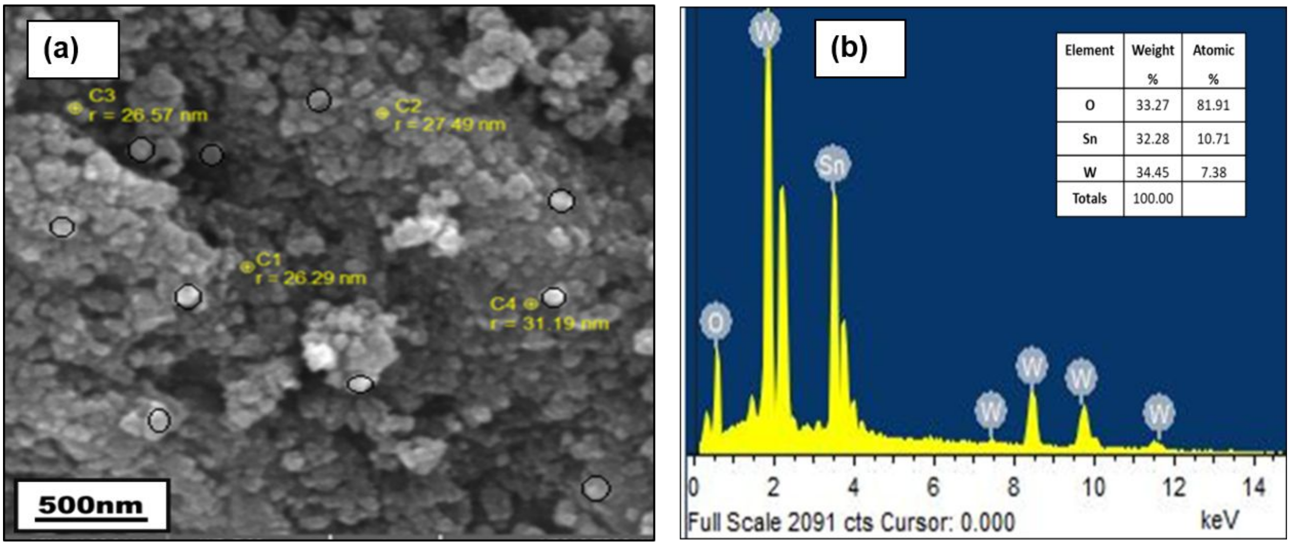

Figure S3. (**a**) SEM micrograph of SnO_2/WO_3 bimetallic NS (**b**) EDS spectrum of SnO_2/WO_3 bimetallic oxide NS.

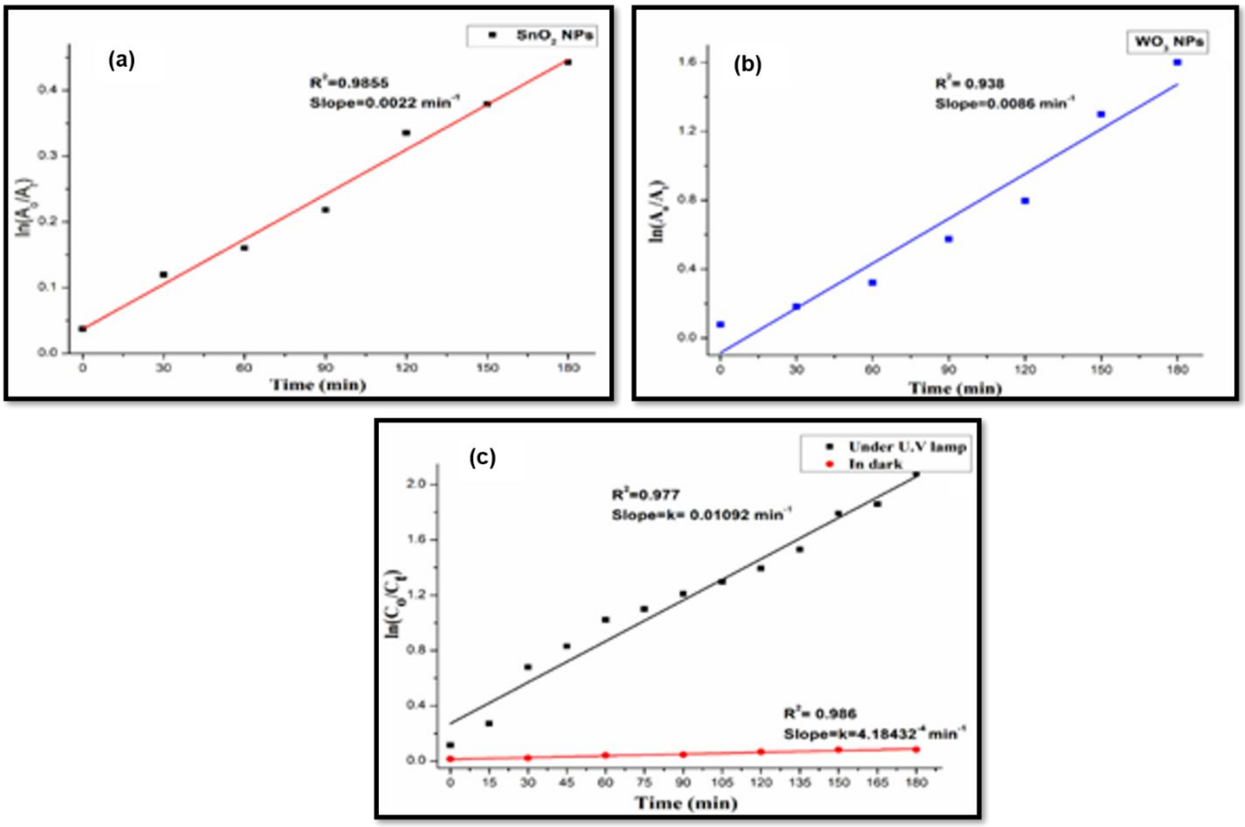

Figure S4. Plot of ln (A_o/A_t) against irradiation time for **(a)** SnO_2 **(b)** WO_3 **(c)** bimetallic photocatalyst.

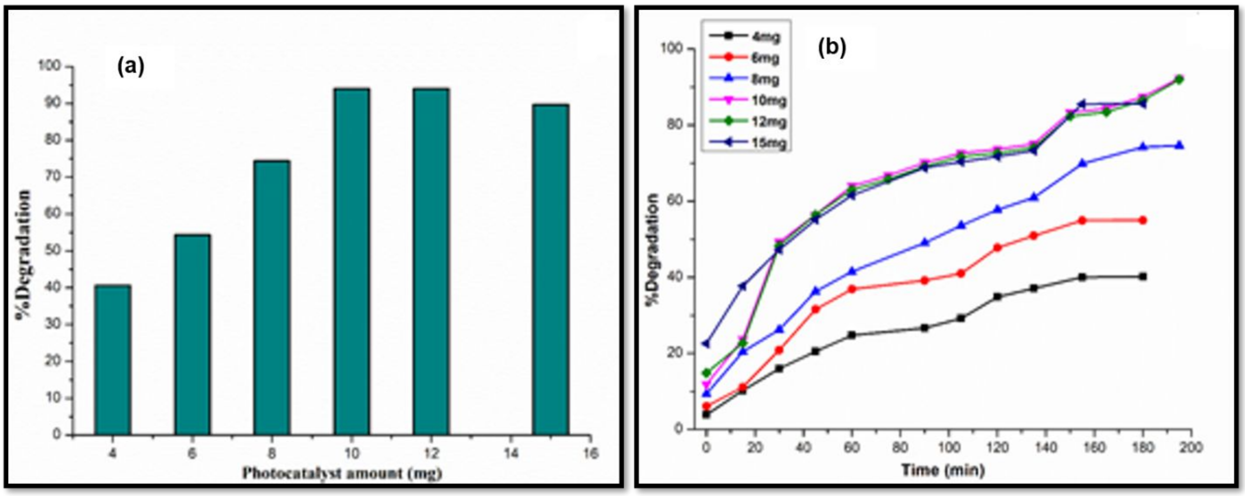

Figure S5. **(a)** Percentage degradation of MB dye (10 ppm) with increasing photocatalyst dose **(b)** Degradation of MB with irradiation time at different photocatalyst doses.

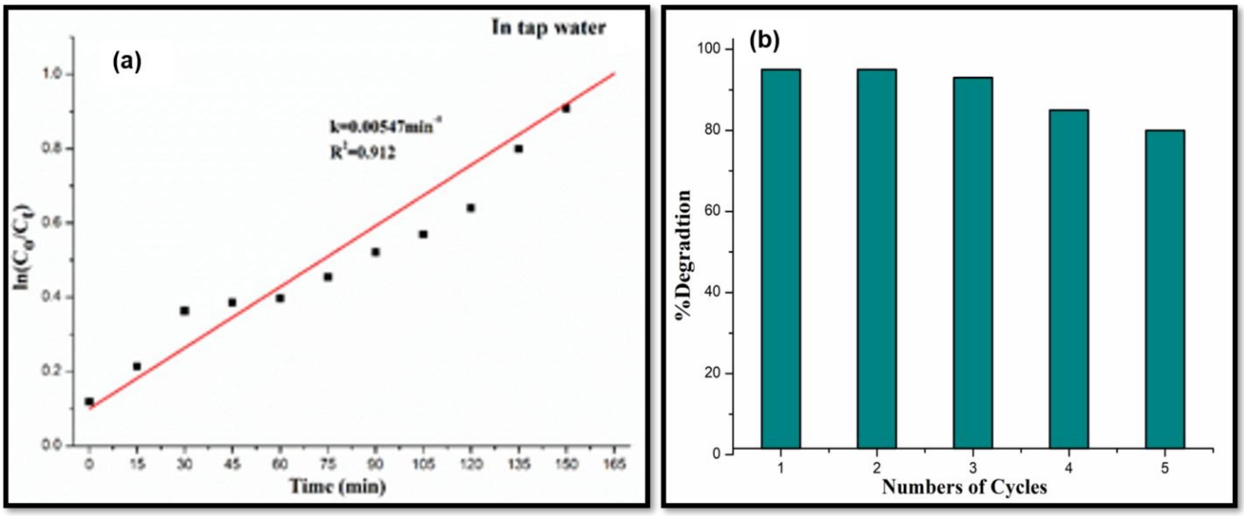

Figure S6. **(a)** Plot of ln (C_o/C_t) versus time in tap water **(b)** Degradation data recorded for reusability of bimetallic oxide photocatalyst up to 5 consecutive cycles.
